# A Genome-Wide Association Study Identified *AFF1* as a Susceptibility Locus for Systemic Lupus Eyrthematosus in Japanese

**DOI:** 10.1371/journal.pgen.1002455

**Published:** 2012-01-26

**Authors:** Yukinori Okada, Kenichi Shimane, Yuta Kochi, Tomoko Tahira, Akari Suzuki, Koichiro Higasa, Atsushi Takahashi, Tetsuya Horita, Tatsuya Atsumi, Tomonori Ishii, Akiko Okamoto, Keishi Fujio, Michito Hirakata, Hirofumi Amano, Yuya Kondo, Satoshi Ito, Kazuki Takada, Akio Mimori, Kazuyoshi Saito, Makoto Kamachi, Yasushi Kawaguchi, Katsunori Ikari, Osman Wael Mohammed, Koichi Matsuda, Chikashi Terao, Koichiro Ohmura, Keiko Myouzen, Naoya Hosono, Tatsuhiko Tsunoda, Norihiro Nishimoto, Tsuneyo Mimori, Fumihiko Matsuda, Yoshiya Tanaka, Takayuki Sumida, Hisashi Yamanaka, Yoshinari Takasaki, Takao Koike, Takahiko Horiuchi, Kenshi Hayashi, Michiaki Kubo, Naoyuki Kamatani, Ryo Yamada, Yusuke Nakamura, Kazuhiko Yamamoto

**Affiliations:** 1 Laboratory for Autoimmune Diseases, Center for Genomic Medicine (CGM), RIKEN, Yokohama, Japan; 2 Department of Allergy and Rheumatology, Graduate School of Medicine, University of Tokyo, Tokyo, Japan; 3 Laboratory for Statistical Analysis, CGM, RIKEN, Yokohama, Japan; 4 Division of Genome Analysis, Research Center for Genetic Information, Medical Institute of Bioregulation, Kyushu University, Fukuoka, Japan; 5 Department of Medicine II, Hokkaido University Graduate School of Medicine, Sapporo, Japan; 6 Department of Hematology and Rheumatology, Tohoku University Graduate School of Medicine, Sendai, Japan; 7 Division of Rheumatology, Department of Internal Medicine, Keio University School of Medicine, Tokyo, Japan; 8 Department of Internal Medicine and Rheumatology, Juntendo University School of Medicine, Tokyo, Japan; 9 Division of Clinical Immunology, Doctoral Program in Clinical Sciences, Graduate School of Comprehensive Human Science, University of Tsukuba, Tsukuba, Japan; 10 Departments of Medicine and Rheumatology, Graduate School, Tokyo Medical and Dental University, Tokyo, Japan; 11 Division of Rheumatic Diseases, National Center for Global Health and Medicine, Tokyo, Japan; 12 First Department of Internal Medicine, University of Occupational and Environmental Health, Kitakyushu, Japan; 13 Department of Immunology and Rheumatology, Unit of Translational Medicine, Graduate School of Biomedical Sciences, Nagasaki University, Nagasaki, Japan; 14 Institute of Rheumatology, Tokyo Women's Medical University, Tokyo, Japan; 15 Laboratory of Molecular Medicine, Human Genome Center, Institute of Medical Science, University of Tokyo, Tokyo, Japan; 16 Department of Rheumatology and Clinical immunology, Graduate School of Medicine, Kyoto University, Kyoto, Japan; 17 Center for Genomic Medicine, Kyoto University Graduate School of Medicine, Kyoto, Japan; 18 Laboratory for Genotyping Development, CGM, RIKEN, Yokohama, Japan; 19 Laboratory for Medical Informatics, CGM, RIKEN, Yokohama, Japan; 20 Laboratory of Immune Regulation, Wakayama Medical University, Wakayama, Japan; 21 Department of Medicine and Biosystemic Science, Kyushu University Graduate School of Medical Sciences, Fukuoka, Japan; University of Oxford, United Kingdom

## Abstract

Systemic lupus erythematosus (SLE) is an autoimmune disease that causes multiple organ damage. Although recent genome-wide association studies (GWAS) have contributed to discovery of SLE susceptibility genes, few studies has been performed in Asian populations. Here, we report a GWAS for SLE examining 891 SLE cases and 3,384 controls and multi-stage replication studies examining 1,387 SLE cases and 28,564 controls in Japanese subjects. Considering that expression quantitative trait loci (eQTLs) have been implicated in genetic risks for autoimmune diseases, we integrated an eQTL study into the results of the GWAS. We observed enrichments of cis-eQTL positive loci among the known SLE susceptibility loci (30.8%) compared to the genome-wide SNPs (6.9%). In addition, we identified a novel association of a variant in the AF4/FMR2 family, member 1 (*AFF1*) gene at 4q21 with SLE susceptibility (rs340630; *P* = 8.3×10^−9^, odds ratio = 1.21). The risk A allele of rs340630 demonstrated a cis-eQTL effect on the *AFF1* transcript with enhanced expression levels (*P*<0.05). As *AFF1* transcripts were prominently expressed in CD4^+^ and CD19^+^ peripheral blood lymphocytes, up-regulation of *AFF1* may cause the abnormality in these lymphocytes, leading to disease onset.

## Author Summary

Although recent genome-wide association study (GWAS) approaches have successfully contributed to disease gene discovery, many susceptibility loci are known to be still uncaptured due to strict significance threshold for multiple hypothesis testing. Therefore, prioritization of GWAS results by incorporating additional information is recommended. Systemic lupus erythematosus (SLE) is an autoimmune disease that causes multiple organ damage. Considering that abnormalities in B cell activity play essential roles in SLE, prioritization based on an expression quantitative trait loci (eQTLs) study for B cells would be a promising approach. In this study, we report a GWAS and multi-stage replication studies for SLE examining 2,278 SLE cases and 31,948 controls in Japanese subjects. We integrated eQTL study into the results of the GWAS and identified *AFF1* as a novel SLE susceptibility loci. We also confirmed cis-regulatory effect of the locus on the *AFF1* transcript. Our study would be one of the initial successes for detecting novel genetic locus using the eQTL study, and it should contribute to our understanding of the genetic loci being uncaptured by standard GWAS approaches.

## Introduction

Systemic lupus erythematosus (SLE) is an autoimmune disease characterized by autoantibody production, complement activation, and multi-organ damage [1]. Familial aggregation demonstrates that both genetic and environmental factors play a role in pathogenesis of SLE [2]. Genetic studies using candidate gene-approaches, and recently, genome-wide association studies (GWAS), have uncovered more than 25 SLE susceptibility genes, including *HLA-DRB1*, *IRF5*, *STAT4*, *ITGAM*, *BLK*, *TNFAIP3*, and others [3]–[18]. However, most of these studies were conducted in European populations [3]–[13], [15], [17], and few studies have been conducted in Asian populations [14], [16], [18]. Since the epidemiology of SLE has demonstrated that the prevalence of disease substantially differs among populations, genetic backgrounds of SLE should be also heterogeneous across populations [19], [20]. Therefore, additional studies in Asians might provide novel insights. It is of note that GWAS for SLE in Chinese populations identified novel loci that had not been detected in Europeans, such as *ETS1*, *IKZF1*, and *WDFY4* [14], [16].

Another issue raised by the previous GWASs for complex diseases is that many susceptibility loci still remained uncaptured, owing to its strict significance threshold for multiple hypothesis testing [21]. In SLE, for example, the 26 risk loci identified by the previous GWAS explained only an estimated 8% of the total genetic susceptibility to the disease [15]. Therefore, it is still important to examine the sub-loci of GWAS, in order to reveal the entire picture of genetic etiology. To effectively explore these uncaptured loci, prioritization of GWAS results by incorporating additional information implicated in the disease pathophysiology is recommended [22], [23]. Considering that abnormalities in B cell activity play essential roles in SLE [1] and that expression quantitative trait loci (eQTL) have been implicated to comprise approximately a half of genetic risks for autoimmune diseases [24], prioritization based on an eQTL study for B cells would be a promising approach for SLE [25]. Moreover, an eQTL itself assures the presence of functional variant(s) that regulate gene expression. Thus, eQTL increases the prior probability of the presence of disease-causal variant(s) in the locus more effectively and unbiasedly, compared to other knowledge-based prioritizations such as gene pathway analysis [24].

Here, we report a GWAS and multi-stage replication studies for SLE examining 2,278 SLE cases and 31,948 controls in Japanese subjects. We integrated eQTL study into the results of the GWAS, which effectively enabled to detect a novel SLE susceptibility locus.

## Results

### GWAS for SLE

**Figure 1:**
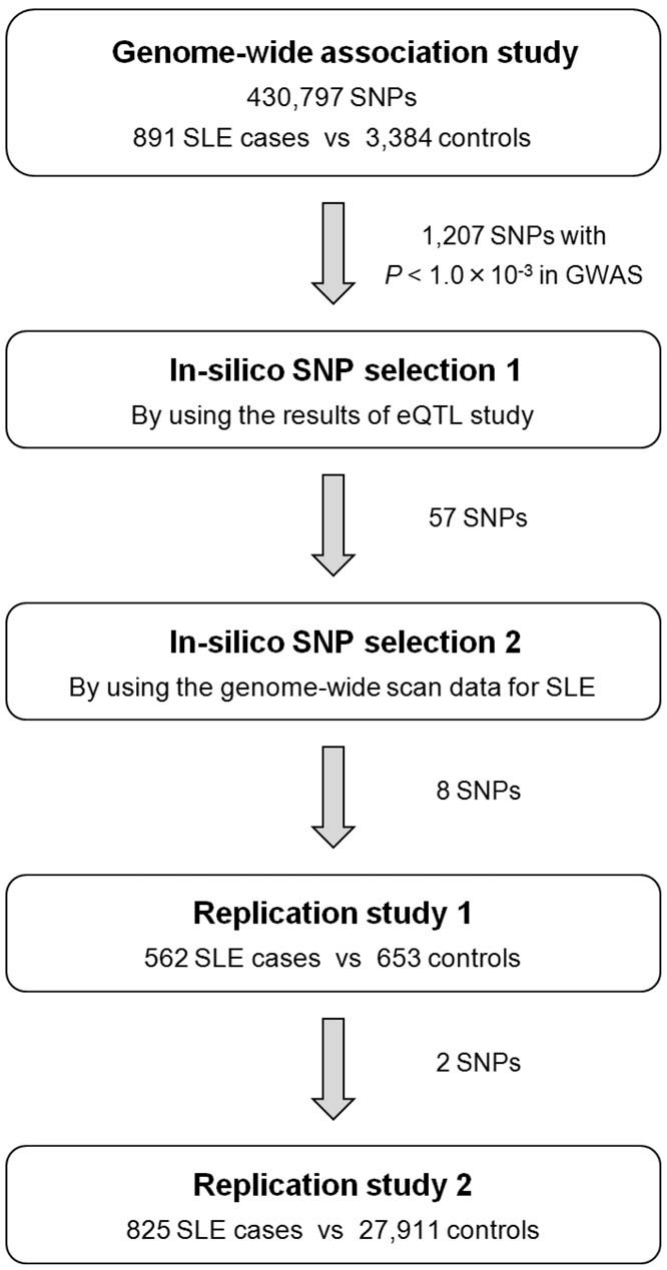
Design of the GWAS and multi-stage replication studies for SLE in Japanese subjects. A total of 2,278 SLE cases and 31,948 controls were enrolled. The clinical characteristics of the subjects are summarized in Table S1 and S2. Details of the genome-wide scan data for SLE referenced in the *in silico* SNP selection 2 are described elsewhere (Tahira T et al. Presented at the 59th Annual Meeting of the American Society of Human Genetics, October 21, 2009).

In the GWAS, 891 SLE cases and 3,384 controls in Japanese subjects were genotyped over 550,000 single nucleotide polymorphism (SNP) markers (Table S1, S2 and Figure 1). We applied stringent quality control (QC) criteria and evaluated associations of 430,797 autosomal SNPs, as previously described [26]. No substantial population stratification was demonstrated through principal component analysis (Figure S1) or a Quantile–Quantile plot of *P*-values (inflation factor, λ_GC_, = 1.088, Figure S2), suggesting homogenous ancestries of our study population [27].

**Figure 2:**
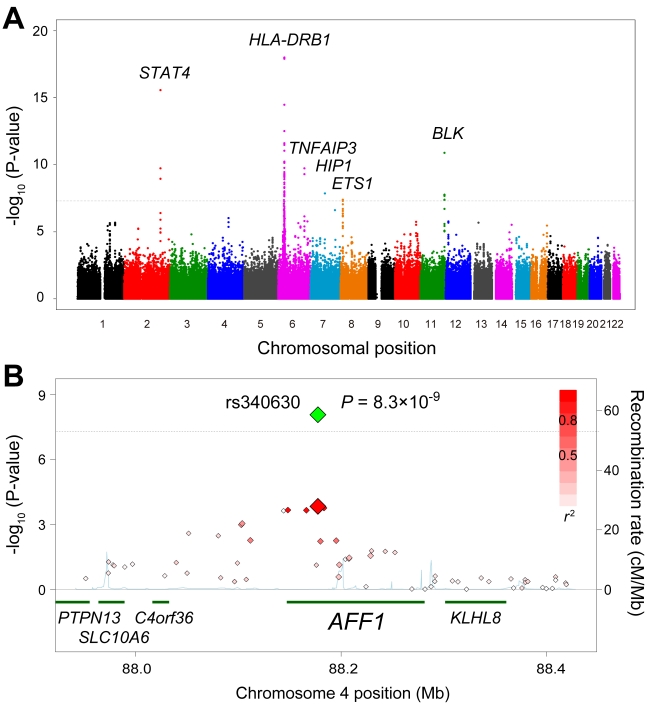
Associations of the *AFF1* locus with SLE. (A) A chromosomal plot of *P*-values in GWAS for SLE. (B) A regional plot in the *AFF1* locus. Diamond-shaped data points represent −log_10_ (*P*-values) of the SNPs. Large-sized points indicate the *P*-values of the landmark SNP, rs340630 (green for the combined study and red for the GWAS). Density of red color represents *r^2^* values with rs340630. Blue line represents recombination rates. Lower part indicates RefSeq genes. Gray dashed horizontal lines represent the threshold of *P* = 5.0×10^−8^. The plots were drawn using SNAP, version 2.1 [47].

We identified significant associations in six chromosomal loci that satisfied the genome-wide significance threshold of *P*<5.0×10^−8^ (Table 1 and Figure 2A). These loci have been reported to be associated with SLE susceptibility (*STAT4*, *TNFAIP3*, *HIP1*, *BLK*, *ETS1*, and the HLA region) [3]–[18]. We also observed significant replications in 17 of the previously reported SLE susceptibility loci [3]–[18] (α = 0.01; Table 2). Of these, significant replications were enriched in the loci identified through the studies in Asian populations (80%; 8 of the 10 loci), including *RASGRP3*, *IKZF1*, *HIP1*, *WDFY4*, intergenic region at 11q23, *ETS1*, *SLC15A4*, *ELF1*, and *HIC2-UBE2L3* [14], [16], [18], compared to those in European populations (56.3%; 9 of the 16 loci) [3]–[13], [15], [17].

**Table 1:** Results of a genome-wide association study for Japanese patients with SLE.

rsID^a^	Chr	Position (bp)	Cytoband	Gene	Allele^b^	No. subjects		Allele 1 freq.		OR (95%CI)	*P*
					1/2	Case	Control	Case	Control		
rs10168266	2	191,644,049	2q32	*STAT4*	T/C	891	3,384	0.37	0.27	1.59 (1.42–1.78)	2.7×10^−16^
rs9501626	6	32,508,322	6p21	HLA region	A/C	891	3,381	0.20	0.12	1.86 (1.62–2.13)	1.0×10^−18^
rs2230926	6	138,237,759	6q23	*TNFAIP3*	G/T	891	3,377	0.11	0.069	1.75 (1.47–2.08)	1.9×10^−10^
rs6964720	7	75,018,280	7q11	*HIP1*	G/A	891	3,384	0.25	0.19	1.43 (1.27–1.63)	1.3×10^−8^
rs2254546	8	11,381,089	8p23	*BLK*	G/A	891	3,384	0.78	0.72	1.42 (1.61–1.25)	4.1×10^−8^
rs6590330	11	127,816,269	11q24	*ETS1*	A/G	891	3,368	0.48	0.39	1.44 (1.30–1.60)	1.3×10^−11^

a SNPs that satisfied the threshold of *P*<5.0×10^−8^ were indicated.

b Based on forward strand of NCBI Build 36.3.

SLE, systemic lupus erythematosus; OR, odds ratio.

**Table 2:** Associations among previously reported SLE-related loci.

rsID	Chr	Position (bp)	Cytoband	Gene	Allele^a^	Allele 1 freq.		OR (95%CI)	*P*	eQTL^b^	Identified by the studies in^c^	
					1/2	Case	Control				Caucasians	Asians
rs2205960	1	171,458,098	1q25	*TNFSF4*	T/G	0.23	0.18	1.35 (1.19–1.54)	3.0×10^−6^		+	
rs3024505	1	205,006,527	1q32	*IL10*	A/G	0.019	0.014	1.34 (0.90–2.00)	0.15		+	
rs13385731	2	33,555,394	2p22	*RASGRP3*	C/T	0.90	0.87	1.37 (1.15–1.64)	6.0×10^−4^	+		+
rs10168266	2	191,644,049	2q32	*STAT4*	T/C	0.37	0.27	1.59 (1.42–1.78)	2.7×10^−16^		+	
rs6445975	3	58,345,217	3p14	*PXK*	G/T	0.25	0.23	1.09 (0.96–1.23)	0.18	+	+	
rs10516487	4	102,970,099	4q24	*BANK1*	G/A	0.91	0.89	1.28 (1.07–1.53)	0.0070		+	
rs10036748	5	150,438,339	5q33	*TNIP1*	T/C	0.75	0.72	1.16 (1.03–1.31)	0.014			+
rs9501626	6	32,508,322	6p21	*HLA-DRB1*	A/C	0.20	0.12	1.86 (1.62–2.13)	1.0×10^−18^		+	
rs548234	6	106,674,727	6q21	*PRDM1*	C/T	0.40	0.34	1.30 (1.16–1.44)	2.3×10^−6^	+	+	
rs2230926	6	138,237,759	6q23	*TNFAIP3*	G/T	0.11	0.069	1.75 (1.47–2.08)	1.9×10^−10^	+	+	
rs849142	7	28,152,416	7p15	*JAZF1*	C/T	0.999	0.999	2.72 (0.25–29.8)	0.41		+	
rs4917014	7	50,276,409	7p12	*IKZF1*	T/G	0.58	0.53	1.24 (1.11–1.38)	8.1×10^−5^			+
rs6964720	7	75,018,280	7q11	*HIP1*	G/A	0.25	0.19	1.43 (1.27–1.62)	1.3×10^−8^			+
rs4728142	7	128,361,203	7q32	*IRF5*	A/G	0.16	0.11	1.48 (1.28–1.72)	2.4×10^−7^	+	+	
rs2254546	8	11,381,089	8p23	*BLK*	G/A	0.78	0.72	1.42 (1.25–1.61)	4.1×10^−8^	+	+	
rs1913517	10	49,789,060	10q11	*WDFY4*	A/G	0.32	0.28	1.20 (1.07–1.35)	0.0013			+
rs4963128	11	579,564	11p15	*KIAA1542*	T/C	0.98	0.97	1.58 (1.03–2.44)	0.038	+	+	
rs2732552	11	35,041,168	11p13	*PDHX*, *CD44*	T/C	0.75	0.73	1.13 (1.00–1.27)	0.056		+	
rs4639966	11	118,078,729	11q23	Intergenic	T/C	0.32	0.28	1.22 (1.09–1.36)	7.3×10^−4^			+
rs6590330	11	127,816,269	11q24	*ETS1*	A/G	0.48	0.39	1.44 (1.30–1.60)	1.3×10^−11^			+
rs1385374	12	127,866,647	12q24	*SLC15A4*	T/C	0.19	0.16	1.21 (1.06–1.38)	0.0057			+
rs7329174	13	40,456,110	13q14	*ELF1*	G/A	0.30	0.25	1.32 (1.18–1.49)	2.2×10^−6^			+
rs7197475	16	30,550,368	16p11	Intergenic	T/C	0.12	0.10	1.20 (1.02–0.41)	0.031			+
rs11150610	16	31,241,737	16p11	*ITGAM*	C/A	0.20	0.19	1.07 (0.94–1.22)	0.32	+	+	
rs12949531	17	13,674,531	17p12	Intergenic	T/C	0.28	0.27	1.02 (0.91–1.15)	0.73		+	
rs463426	22	20,139,185	22q11	*HIC2,UBE2L3*	T/C	0.52	0.48	1.20 (1.08–1.33)	6.1×10^−4^		+	

a Based on forward strand of NCBI Build 36.3.

b Defined using gene expression data measured in lymphoblastoid B cell lines [28].

c Based on the previously reported studies for SLE susceptibility loci [3]–[18].

SLE, systemic lupus erythematosus; OR, odds ratio; eQTL, expression quantitative trait locus; GWAS, genome-wide association study.

### Incorporation of eQTL study into GWAS results

For the selection of SNPs incorporated in the replication studies of the potential association signals, we evaluated cis-eQTL effects of the SNPs using publically available gene expression data [28], and prioritized the results of the GWAS. After applying QC criteria, we evaluated the expression levels of 19,047 probes assayed in lymphoblastoid B cell lines from Phase II HapMap East-Asian individuals [29] using Illumina's human whole-genome expression array (WG-6 version 1) [28]. For each of the SNPs included in our GWAS, probes located within ±300 kbp regions were focused on as cis-eQTLs (average 4.93 probes per SNP). We denoted the SNPs which exhibited significant associations with expression levels of any of the corresponding cis-eQTLs as eQTL positive (false discovery rate (FDR) *Q*-values<0.2). We observed enrichments of eQTL positive loci among the SLE susceptibility loci (30.8%; 8 of the 26 evaluated loci) including a well-known eQTL gene of *BLK* [11], [25] (Table 2), compared to the genome-wide SNPs (6.9%) and compared even to the SNPs in the vicinity of expressed loci (among the SNPs located within ±10 kbp of probes used for the expression analysis, 13.1% were eQTL positive; Table S3).

By prioritizing the results of the GWAS using the eQTL study, we selected 57 SNPs from 1,207 SNPs that satisfied *P*<1.0×10^−3^ in the GWAS. We subsequently referred the associations of the selected SNPs using the results of the concurrent genome-wide scan for SLE in an independent Japanese population (Tahira T et al. Presented at the 59th Annual Meeting of the American Society of Human Genetics, October 21, 2009). In the scan, 447 SLE cases and 680 controls of Japanese origin were evaluated using a pooled DNA approach [30]. We selected SNPs if any association signals were observed in the neighboring SNPs of the pooled analysis. As a result, 8 SNPs remained for further investigation (Table S4).

### Replication studies and identification of *AFF1*

Then, we performed two-stage replication studies using independent SLE cohorts for Japanese subjects (cohort 1 with 562 SLE cases and 653 controls, and cohort 2 with 825 SLE cases and 27,911 controls). First, we evaluated the selected 8 SNPs in the replication study 1. In the replication study 2, 2 SNPs that satisfied *P*<1.0×10^−6^ in the combined study of GWAS and replication study 1 were further evaluated (Figure 1). Among the evaluated SNPs, we observed significant replications in the SNP located in the genomic region of the AF4/FMR2 family, member 1 gene (*AFF1*) at 4q21 (rs340630; *P* = 4.6×10^−5^ and *P* = 0.0094 in the two individual cohorts, respectively; Table 3, Table S5, and Figure 2B). The combined study for the GWAS (*P* = 1.5×10^−4^) and the replication studies demonstrated significant associations of rs340630 that satisfied the genome-wide significance threshold (*P* = 8.3×10^−9^, OR = 1.21, 95% CI 1.14–2.30).

**Table 3:** Results of combined study for Japanese patients with SLE.

rsID	Chr	Position (bp)	Cytoband	Gene	Allele	Stage	No. subjects		Allele 1 freq.		OR (95%CI)	*P*	eQTL^a^
					1/2		Case	Control	Case	Control			
rs340630	4	88,177,419	4q21	*AFF1*	A/G	GWAS	891	3,383	0.56	0.51	1.22 (1.10–1.36)	1.5×10^−4^	+
						Replication study 1	550	646	0.57	0.49	1.40 (1.19–1.64)	4.6×10^−5^	
						Replication study 2	820	27,911	0.56	0.53	1.14 (1.03–1.26)	0.0094	
						Combined study	2,261	31,940	0.56	0.52	1.21 (1.14–1.30)	8.3×10^−9^	

a Defined using gene expression data measured in lymphoblastoid B cell lines [28].

### Cis-eQTL effect of rs340630 on *AFF1* transcripts

**Figure 3:**
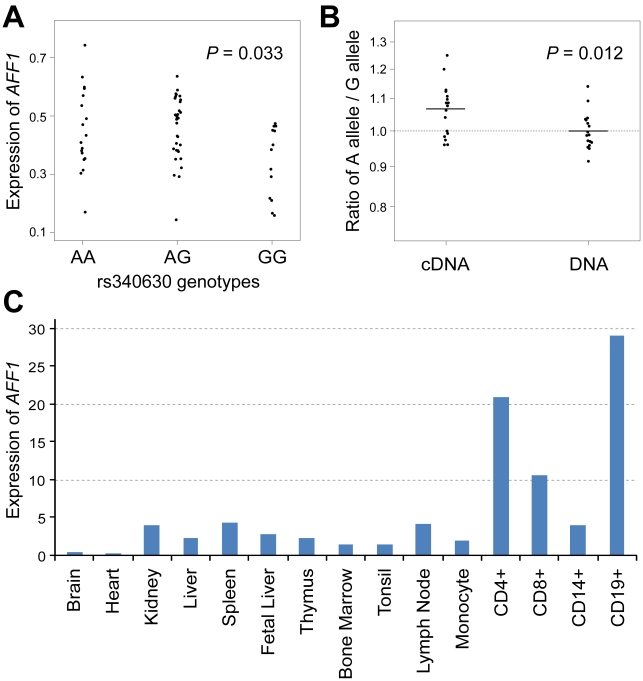
Association of rs340630 with *AFF1* expression. (A) Correlation between rs340630 genotypes and transcript levels of *AFF1* (NM_001166693) in EBV-transfected cell lines (*n* = 62) stimulated with PMA. (B) Allele-specific quantification (ASTQ) of *AFF1* transcripts. Allele specific-probes for rs340638 were used for quantification by qPCR. The ratios of A allele over G allele for the amounts of both cDNAs and DNAs were plotted in log scale for each cell line. (C) *AFF1* expression in various tissues. Transcripts levels of *AFF*1 were quantified by qPCR and were normalized by *GAPDH* levels.

Since the landmark SNP in the *AFF1* locus, rs340630, was prioritized through the eQTL study as an eQTL positive SNP (Table 3), we further validated its cis-eQTL effect using Epstein-Barr virus (EBV)-transfected B cell lines established from Japanese individuals (Pharma SNP Consortium (PSC) cells, *n* = 62). The correlation between rs340630 genotypes and the expression levels of *AFF1* was significant in the PSC cells stimulated with phorbol myristate acetate (PMA) (*R^2^* = 0.074, *P* = 0.033; Figure 3A). The expression levels increased with the number of SLE-risk (A) alleles. To further confirm this cis-regulatory effect, we performed allele-specific transcript quantification (ASTQ) of *AFF1*. The transcript levels of each allele were quantified by qPCR using an allele specific probe for a SNP in the 5′-untranslated region (rs340638), which was in absolute LD with rs340630 (*r^2^* = 1.0, *D′* = 1.0). We examined PSC-cells (*n* = 17) that were heterozygous for both rs340630 and rs340638. The mean ratio of each transcript (A over G allele; the A allele comprises a haplotype with the risk (A) allele of rs340630) were significantly increased to 1.07 compared to the ratio of the amount of DNA (1.00, *P* = 0.012) (Figure 3B). These results suggest that rs340630, or SNP(s) in LD with it, are a regulatory variant predisposing SLE susceptibility through increased expression levels of *AFF1*.

### Expression of *AFF1* in CD4^+^ and CD19^+^ peripheral blood lymphocytes

*AFF1* is known to be involved in cytogenetic translocations of acute lymphoblastic leukemia (ALL) [31]. Its fusion protein with the mixed-lineage leukemia gene (*MLL*) is implicated in the regulation of transcription and the cell cycle of lymphocytes [31]. To investigate the expression pattern of *AFF1* in normal tissues, we evaluated the transcript levels of *AFF1* in a panel of various tissues. We observed prominent expression of *AFF1* in CD4^+^ and CD19^+^ peripheral blood lymphocytes, implying an important role for *AFF1* in helper-T-cells and B-cells (Figure 3C).

## Discussion

Through a GWAS and multi-staged replication studies consisting of 2,278 SLE cases and 31,948 controls in Japanese subjects, our study identified that the *AFF1* locus was significantly associated with SLE susceptibility.

As well as the identification of the novel SLE susceptibility locus, we observed significant replications of associations in the previously reported susceptibility loci. The replications were especially enriched in the loci identified through the studies in Asian populations, compared to those in European populations. Considering the ethnical heterogeneities in the epidemiology of SLE [19], [20], these observations suggest the similarities in the genetic backgrounds of SLE shared within Asian populations, and also the existence of the both common and divergent genetic backgrounds encompassed between European and Asian populations.

To effectively detect the novel SLE susceptibility locus, we integrated cis-eQTL effects of the SNPs and prioritized the results of the GWAS. In addition to identifying a novel locus for SLE-susceptibility, our study demonstrated approximately 30% of confirmed SLE-susceptibility loci were comprised of cis-eQTLs. We also confirmed cis-regulatory effect of the landmark SNP in the *AFF1* locus, rs340630, on *AFF1* transcripts, which had been prioritized through the eQTL study. These results would suggest that accumulation of quantitative changes in gene expression would accelerate the disease onset of SLE. It would also demonstrate the validity of applying eQTL study in the search of the susceptible genes for SLE or other autoimmune diseases, as previously suggested in the study for celiac disease [24]. To our knowledge, this is one of the initial studies to successfully discover a new locus by prioritizing GWAS results using eQTLs, and should contribute to the approaches assessing genetic loci still being uncaptured by recent large-scaled GWASs due to stringent significance threshold for multiple hypothesis testing [21].

We observed prominent expression levels of *AFF1* in CD4^+^ and CD19^+^ peripheral blood lymphocytes, which would imply an important role for *AFF1* in helper-T-cells and B-cells. In fact, *AFF1* is essential for normal lymphocyte development, as demonstrated in mice deficient for *AFF1*; severe reduction were observed in the thymic double positive CD4/CD8 population and the bone marrow pre-B and mature B-cell numbers [32]. The risk A allele of rs340630 demonstrated a cis-eQTL effect on the *AFF1* transcript with enhanced expression levels. As the *AFF1* locus was also demonstrated as an eQTL in primary liver cells [33], the cis-regulatory effect may hold in primary cells as well as lymphoblastoid cells used in the present study. However, because the mechanism of transcriptional regulation is substantially different among cell types [34], cell-type specific analyses including those for primary T-cells and B-cells are needed for understanding the precise role of *AFF1* variant in primary lymphocytes. Although further functional investigation is necessary, our observation suggested that *AFF1* is involved in the etiology of SLE through the regulation of development and activity of lymphocytes. It is of note that *AFF3*, which also belongs to the AF4/FMR2 family, is associated with susceptibility to autoimmune diseases [35].

One of our study's limitations is the selection of SNPs for the replication study using the results of the pooled DNA approach [30], which used a different genotyping platform from that of the present GWAS. Moreover, the association signals based on Silhouette scores in pooled analysis would be less reliable compared to those based on individual genotyping. Since direct comparisons of the association signals of the same single SNPs between the studies would be difficult due to these issues, we adopted the complementary approach that referred the association signals of the multiple SNPs in the pooled analysis for each of the single SNPs in the GWAS, taking account of LD and physical distances between the SNPs. However, there would exist a possibility that the variant(s) truly associated with SLE was left not to be examined in the replication study. It should be noted that only 1 SNP among the 8 selected SNPs yielded the significant association with SLE, although further enrichments of the significant associations might be anticipated. To elucidate effectiveness and limitation of our approach, further assessments of the studies on the remaining loci would be desirable. It should also be noted that the control-case ratio of the subjects were relatively high in the replication study 2 ( = 33.8), and this disproportionate ratio could have induced potential bias on the results of the association analysis of the SNPs. However, considering the homogeneous ancestries of the Japanese population [27] and that principal component analysis did not demonstrate significant population stratification in the control subjects of the replication study 2 (data not shown), the bias owing to population stratification might not be substantial.

In summary, through a GWAS and multi-staged replication studies in a Japanese population integrating eQTL study, our study identified *AFF1* as a novel susceptibility locus for SLE.

## Materials and Methods

### Subjects

We enrolled 2,278 systemic lupus erythematosus (SLE) cases and 31,948 controls. SLE cases enrolled in the genome-wide association study (GWAS) (*n* = 891) or part of the 2nd replication study (*n* = 83) were collected from 12 medical institutes in Japan under the support of the autoimmune disease study group of Research in Intractable Diseases, Japanese Ministry of Health, Labor and Welfare: Hokkaido University Graduate School of Medicine, Tohoku University Graduate School of Medicine, the University of Tokyo, Keio University School of Medicine, Juntendo University School of Medicine, University of Occupational and Environmental Health, University of Tsukuba, Tokyo Medical and Dental University, National Center for Global Health and Medicine, Nagasaki University, Wakayama Medical University, and Jichi Medical University. SLE cases (*n* = 562) and controls (*n* = 653) enrolled in the 1st replication study were collected from Kyushu University. Some of the SLE cases (*n* = 742) and controls (*n* = 27,911) enrolled in the 2nd replication study were collected from Kyoto University, Tokyo Women's Medical University, the University of Tokyo, and the BioBank Japan Project [36]. All subjects were of Japanese origin and provided written informed consent. SLE cases met the revised American College of Rheumatology (ACR) criteria for SLE [37]. Control subjects were confirmed to be free of autoimmune disease. Some of the SLE cases were included in our previous studies [38]–[40]. Details of the subjects are summarized in Table S1 and S2. This research project was approved by the ethical committees of the University of Tokyo, RIKEN, and affiliated medical institutes.

### Genotyping and quality control

In GWAS, 946 SLE cases and 3,477 controls were genotyped using Illumina HumanHap610-Quad and Illumina HumanHap550v3 Genotyping BeadChips (Illumina, CA, USA), respectively. After the exclusion of 47 SLE cases and 92 controls with call rates <0.98, SNPs with call rates <0.99 in SLE cases or controls, non-autosomal SNPs, and SNPs not shared between SLE cases and controls, were excluded. We excluded 7 closely related SLE cases in a 1st or 2nd degree of kinship based on identity-by-descent estimated using PLINK version 1.06 [41]. We then excluded 1 SLE cases and 1 controls whose ancestries were estimated to be distinct from East-Asian populations using PCA performed along with the genotype data of Phase II HapMap populations (release 24) [29] using EIGENSTRAT version 2.0 [42]. Subsequently, SNPs with minor allele frequencies <0.01 in SLE cases or controls, SNPs with exact *P*-values of Hardy-Weinberg equilibrium test <1.0×10^−6^ in controls, or SNPs with ambiguous cluster plots were excluded. Finally, 430,797 SNPs for 891 SLE cases and 3,384 controls were obtained. Genotyping of SNPs in replication studies was performed using TaqMan Assay or Illumina HumanHap610-Quad Genotyping BeadChip (Illumina, CA, USA).

### Association analysis of the SNPs

Association of SNPs in GWAS and replication studies were tested with Cochran-Armitage's trend test. Combined analysis was performed with Mantel-Haenzel method. Associations of previously reported SLE susceptibility loci [3]–[18] were evaluated using the results of the GWAS. Genotype imputation was performed for non-genotyped SNPs using MACH version 1.0 [43] with Phase II HapMap East-Asian individuals as references [29], as previously described [44]. All imputed SNPs demonstrated imputation scores, *Rsq*, >0.70.

### eQTL study

We analyzed gene expression data previously measured in lymphoblastoid B cell lines from Phase II HapMap East-Asian individuals using Illumina's human whole-genome expression array (WG-6 version 1) (accession number; GSE6536) [28]. Expression data were normalized across the individuals. We used BLAST to map 47,294 Illumina array probes onto human autosomal reference genome sequences (Build 36). We discarded probes mapped with expectation values smaller than 0.01 to multiple loci, or for which there was polymorphic HapMap SNP(s) inside the probe. Then, 19,047 probes with exact matches to a unique locus with 100% identity and with a mean signal intensity greater than background were obtained. Genotype data of HapMap individuals were obtained for SNPs included in the GWAS. Associations of SNP genotypes (coded as 0, 1, and 2) with expression levels of each of the cis-eQTL probes (located within ±300 kbp regions of the SNPs) were evaluated using linear regression assuming additive effects of the genotypes on the expression levels. Considering the significant overlap between eQTL and genetic loci responsible for autoimmune diseases [24], we applied relatively less stringent multiple testing threshold of FDR *Q*-values<0.2 for the definition of eQTL. SNPs that exhibited this threshold with any of the corresponding cis-eQTL probes were denoted as eQTL positive.

### Selection of SNPs enrolled in the replication studies

In order to select SNPs for further replication studies, we firstly integrated the results of GWAS and eQTL study. SNPs that satisfied *P*<1.0×10^−4^ in GWAS, or the SNPs that satisfied 1.0×10^−4^≤*P*<1.0×10^−3^ in GWAS and denoted as eQTL positive, were selected. Among these, SNPs most significantly associated in each of the genomic loci and not included in the previously reported SLE susceptibility loci [3]–[18] were further evaluated.

Then, the results of the concurrently proceeding genome-wide scan for SLE in the Japanese subjects using a pooled DNA approach were referred (Tahira T et al. Presented at the 59th Annual Meeting of the American Society of Human Genetics, October 21, 2009). In the scan, DNA collected from 447 SLE cases and 680 controls of Japanese origin were pooled respectively, and genotyped using GeneChip Human Mapping 500K Array Set (Affymetrix, CA, USA). SNPs were ranked according to the Silhouette scores estimated based on relative allele scores (RAS) between SLE cases and controls, and rank-based *P*-values were assigned [30]. By referring to association signals in multiple neighboring SNPs in the pooled analysis, we selected SNPs for replication study 1. Namely, if the SNP of interest was in LD (*r^2^*>0.5) or was located within ±100 kbp of SNPs showing association signals in the pooled analysis (rank-based *P*<0.01), it would be selected. SNPs that satisfied *P*<1.0×10^−6^ in the combined study of GWAS and replication study 1 were further evaluated in replication study 2 (Figure 1).

### Quantification of *AFF1* expression

EBV-transformed lymphoblastoid cell lines (*n* = 62) were established by Pharma SNP Consortium (Tokyo, Japan) using peripheral blood lymphocytes of Japanese healthy individuals. Cells were incubated for 2 h in medium alone (RPMI 1640 medium containing 10% FBS, 1% penicillin, and 1% streptomycin) or with 100 ng/ml PMA. Conditions for cell stimulation were optimized before the experiment as previously described [45]. Cells were then harvested and total RNA was isolated using an RNeasy Mini Kit (Qiagen) with DNase treatment. Total RNA (1 µg) was reverse transcribed using TaqMan Gold RT-PCR reagents with random hexamers (Applied Biosystems). Real-time quantitative PCR was performed in triplicate using an ABI PRISM 7900 and TaqMan gene expression assays (Applied Biosystems). Specific probes (Hs01089428_m1) for transcript of *AFF1* (NM_001166693) were used. Expression of *AFF1* in various tissues was also quantified using Premium Total RNA (Clontech). The data were normalized to *GAPDH* levels. *GUS* levels were also evaluated for internal control, and similar results were obtained. Correlation coefficient, *R^2^*, between rs340630 genotypes and transcript levels of *AFF1* was evaluated.

### Allele-specific transcript quantification (ASTQ)

ASTQ of *AFF1* in PSC cells was performed as previously described [46]. DNAs were extracted by using a DNeasy Kit (QIAGEN). RNA extraction and cDNA preparation were performed as described above. For PSC cells (*n* = 17) that were heterozygous for both rs340630 (the landmark SNP of GWAS) and rs340638 (located in the 5′-untranslated region of *AFF1* and in absolute LD with rs340630), expression levels of *AFF1* were quantified by qPCR on an ABI Prism 7900 using a custom-made TaqMan MGB-probe set for rs340638. Primer sequences were 5′-CTAACTGTGGCCCGCGTTG-3′ and 5′-CCCGGCGCAGTTTCTGAG-3′. The probe sequences were 5′-VIC-CGAAGACCGCCAGCGCCCAAC-TAMRA-3′ and 5′-FAM-CGAAGACCGCCGGCGCCCAA-TAMRA-3′. Ct values of VIC and FAM were obtained for genomic DNA and cDNA samples after 40 cycles of real-time PCR. We also prepared genomic DNA of samples homozygous for each allele and mixed them at different ratios (2∶8, 3∶7, 4∶6, 5∶5, 6∶4, 7∶3, 8∶2) to create a standard curve by plotting Ct values of VIC/FAM against the allelic ratio of VIC/FAM for each mixture. Using the standard curve, we calculated the allelic ratios for each genomic DNA and cDNA samples. We measured each sample in quadruplicate in one assay; tests were independently repeated twice.

### Web resources

The URLs for data presented herein are as follows.

NCBI GEO, http://www.ncbi.nlm.nih.gov/geo

BioBank Japan Project, http://biobankjp.org

PLINK software, http://pngu.mgh.harvard.edu/~purcell/plink/index.shtml

International HapMap Project, http://www.hapmap.org

EIGENSTRAT software, http://genepath.med.harvard.edu/~reich/Software.htm

MACH and mach2qtl software, http://www.sph.umich.edu/csg/abecasis/MACH/index.html

SNAP, http://www.broadinstitute.org/mpg/snap/index.php

## Supporting Information

Figure S1 Principal component analysis (PCA) plot of the subjects. PCA plot of subjects enrolled in the GWAS for SLE. SLE cases and the controls enrolled in the GWAS are plotted based on eigenvectors 1 and 2 obtained from the PCA using EIGENSTRAT version 2.0 [42], along with European (CEU), African (YRI), Japanese (JPT), and Chinese (CHB) individuals obtained from the Phase II HapMap database (release 22) [29]. Subjects who were estimated to be outliers in terms of ancestry from East-Asian (JPT+CHB) clusters and excluded from the study are indicated by black arrows.
(TIF)

Figure S2 Quantile-Quantile plot (QQ-plot) of *P*-values in the GWAS for SLE. The horizontal axis indicates the expected −log_10_ (*P*-values). The vertical axis indicates the observed −log_10_ (*P*-values). The QQ-plot for the *P*-values of all SNPs that passed the quality control criteria is indicated in blue. The QQ-plot for the *P*-values after the removal of SNPs included in the previously reported SLE susceptibility loci is indicated in black. The gray line represents *y* = *x*. The SNPs for which the *P*-value was smaller than 1.0×10^−15^ are indicated at the upper limit of the plot.
(TIF)

Table S1 Basal characteristics of cohorts.
(DOC)

Table S2 Frequency of clinical characteristics of SLE in this GWAS.
(DOC)

Table S3 Distributions of eQTL positivity rates of the SNPs.
(DOC)

Table S4 Results of replication study 1 for Japanese patients with SLE.
(DOC)

Table S5 Results of replication studies 1 and 2 for Japanese patients with SLE.
(DOC)

## References

[1] Lipsky PE (2001). Systemic lupus erythematosus: an autoimmune disease of B cell hyperactivity.. Nat Immunol.

[2] Sestak AL, Shaver TS, Moser KL, Neas BR, Harley JB (1999). Familial aggregation of lupus and autoimmunity in an unusual multiplex pedigree.. J Rheumatol.

[3]  Sigurdsson S, Nordmark G, Goring HH, Lindroos K, Wiman AC (2005). Polymorphisms in the tyrosine kinase 2 and interferon regulatory factor 5 genes are associated with systemic lupus erythematosus.. Am J Hum Genet.

[4] Graham RR, Kozyrev SV, Baechler EC, Reddy MV, Plenge RM (2006). A common haplotype of interferon regulatory factor 5 (IRF5) regulates splicing and expression and is associated with increased risk of systemic lupus erythematosus.. Nat Genet.

[5] Graham RR, Kyogoku C, Sigurdsson S, Vlasova IA, Davies LR (2007). Three functional variants of IFN regulatory factor 5 (IRF5) define risk and protective haplotypes for human lupus.. Proc Natl Acad Sci U S A.

[6] Remmers EF, Plenge RM, Lee AT, Graham RR, Hom G (2007). STAT4 and the risk of rheumatoid arthritis and systemic lupus erythematosus.. N Engl J Med.

[7] Cunninghame Graham DS, Graham RR, Manku H, Wong AK, Whittaker JC (2008). Polymorphism at the TNF superfamily gene TNFSF4 confers susceptibility to systemic lupus erythematosus.. Nat Genet.

[8] Nath SK, Han S, Kim-Howard X, Kelly JA, Viswanathan P (2008). A nonsynonymous functional variant in integrin-alpha(M) (encoded by ITGAM) is associated with systemic lupus erythematosus.. Nat Genet.

[9] Harley JB, Alarcon-Riquelme ME, Criswell LA, Jacob CO, Kimberly RP (2008). Genome-wide association scan in women with systemic lupus erythematosus identifies susceptibility variants in ITGAM, PXK, KIAA1542 and other loci.. Nat Genet.

[10] Kozyrev SV, Abelson AK, Wojcik J, Zaghlool A, Linga Reddy MV (2008). Functional variants in the B-cell gene BANK1 are associated with systemic lupus erythematosus.. Nat Genet.

[11] Hom G, Graham RR, Modrek B, Taylor KE, Ortmann W (2008). Association of systemic lupus erythematosus with C8orf13-BLK and ITGAM-ITGAX.. N Engl J Med.

[12] Graham RR, Cotsapas C, Davies L, Hackett R, Lessard CJ (2008). Genetic variants near TNFAIP3 on 6q23 are associated with systemic lupus erythematosus.. Nat Genet.

[13] Musone SL, Taylor KE, Lu TT, Nititham J, Ferreira RC (2008). Multiple polymorphisms in the TNFAIP3 region are independently associated with systemic lupus erythematosus.. Nat Genet.

[14] Han JW, Zheng HF, Cui Y, Sun LD, Ye DQ (2009). Genome-wide association study in a Chinese Han population identifies nine new susceptibility loci for systemic lupus erythematosus.. Nat Genet.

[15] Gateva V, Sandling JK, Hom G, Taylor KE, Chung SA (2009). A large-scale replication study identifies TNIP1, PRDM1, JAZF1, UHRF1BP1 and IL10 as risk loci for systemic lupus erythematosus.. Nat Genet.

[16] Yang W, Shen N, Ye DQ, Liu Q, Zhang Y (2010). Genome-wide association study in Asian populations identifies variants in ETS1 and WDFY4 associated with systemic lupus erythematosus.. PLoS Genet.

[17] Lessard CJ, Adrianto I, Kelly JA, Kaufman KM, Grundahl KM (2011). Identification of a systemic lupus erythematosus susceptibility locus at 11p13 between PDHX and CD44 in a multiethnic study.. Am J Hum Genet.

[18] Yang J, Yang W, Hirankarn N, Ye DQ, Zhang Y (2011). ELF1 is associated with systemic lupus erythematosus in Asian populations.. Hum Mol Genet.

[19] Hopkinson ND, Doherty M, Powell RJ (1994). Clinical features and race-specific incidence/prevalence rates of systemic lupus erythematosus in a geographically complete cohort of patients.. Ann Rheum Dis.

[20] Danchenko N, Satia JA, Anthony MS (2006). Epidemiology of systemic lupus erythematosus: a comparison of worldwide disease burden.. Lupus.

[21] Yang J, Benyamin B, McEvoy BP, Gordon S, Henders AK (2010). Common SNPs explain a large proportion of the heritability for human height.. Nat Genet.

[22] Raychaudhuri S, Plenge RM, Rossin EJ, Ng AC, Purcell SM (2009). Identifying relationships among genomic disease regions: predicting genes at pathogenic SNP associations and rare deletions.. PLoS Genet.

[23] Cantor RM, Lange K, Sinsheimer JS (2010). Prioritizing GWAS results: A review of statistical methods and recommendations for their application.. Am J Hum Genet.

[24] Dubois PC, Trynka G, Franke L, Hunt KA, Romanos J (2010). Multiple common variants for celiac disease influencing immune gene expression.. Nat Genet.

[25] Cookson W, Liang L, Abecasis G, Moffatt M, Lathrop M (2009). Mapping complex disease traits with global gene expression.. Nat Rev Genet.

[26] Kochi Y, Okada Y, Suzuki A, Ikari K, Terao C (2010). A regulatory variant in CCR6 is associated with rheumatoid arthritis susceptibility.. Nat Genet.

[27] Yamaguchi-Kabata Y, Nakazono K, Takahashi A, Saito S, Hosono N (2008). Japanese population structure, based on SNP genotypes from 7003 individuals compared to other ethnic groups: effects on population-based association studies.. Am J Hum Genet.

[28] Stranger BE, Nica AC, Forrest MS, Dimas A, Bird CP (2007). Population genomics of human gene expression.. Nat Genet.

[29] The International HapMap Consortium (2003). The International HapMap Project.. Nature.

[30] Pearson JV, Huentelman MJ, Halperin RF, Tembe WD, Melquist S (2007). Identification of the genetic basis for complex disorders by use of pooling-based genomewide single-nucleotide-polymorphism association studies.. Am J Hum Genet.

[31] Xia ZB, Popovic R, Chen J, Theisler C, Stuart T (2005). The MLL fusion gene, MLL-AF4, regulates cyclin-dependent kinase inhibitor CDKN1B (p27kip1) expression.. Proc Natl Acad Sci U S A.

[32] Isnard P, Core N, Naquet P, Djabali M (2000). Altered lymphoid development in mice deficient for the mAF4 proto-oncogene.. Blood.

[33] Schadt EE, Molony C, Chudin E, Hao K, Yang X (2008). Mapping the genetic architecture of gene expression in human liver.. PLoS Biol.

[34] Ernst J, Kheradpour P, Mikkelsen TS, Shoresh N, Ward LD (2011). Mapping and analysis of chromatin state dynamics in nine human cell types.. Nature.

[35] Stahl EA, Raychaudhuri S, Remmers EF, Xie G, Eyre S (2010). Genome-wide association study meta-analysis identifies seven new rheumatoid arthritis risk loci.. Nat Genet.

[36] Nakamura Y (2007). The BioBank Japan Project.. Clin Adv Hematol Oncol.

[37] Hochberg MC (1997). Updating the American College of Rheumatology revised criteria for the classification of systemic lupus erythematosus.. Arthritis Rheum.

[38] Suzuki A, Yamada R, Kochi Y, Sawada T, Okada Y (2008). Functional SNPs in CD244 increase the risk of rheumatoid arthritis in a Japanese population.. Nat Genet.

[39] Shimane K, Kochi Y, Horita T, Ikari K, Amano H (2010). The association of a nonsynonymous single-nucleotide polymorphism in TNFAIP3 with systemic lupus erythematosus and rheumatoid arthritis in the Japanese population.. Arthritis Rheum.

[40] Myouzen K, Kochi Y, Shimane K, Fujio K, Okamura T (2010). Regulatory polymorphisms in EGR2 are associated with susceptibility to systemic lupus erythematosus.. Hum Mol Genet.

[41] Purcell S, Neale B, Todd-Brown K, Thomas L, Ferreira MA (2007). PLINK: a tool set for whole-genome association and population-based linkage analyses.. Am J Hum Genet.

[42] Price AL, Patterson NJ, Plenge RM, Weinblatt ME, Shadick NA (2006). Principal components analysis corrects for stratification in genome-wide association studies.. Nat Genet.

[43] Li Y, Willer C, Sanna S, Abecasis G (2009). Genotype imputation.. Annu Rev Genomics Hum Genet.

[44] Okada Y, Takahashi A, Ohmiya H, Kumasaka N, Kamatani Y (2011). Genome-wide association study for C-reactive protein levels identified pleiotropic associations in the IL6 locus.. Hum Mol Genet.

[45] Aikawa Y, Yamamoto M, Yamamoto T, Morimoto K, Tanaka K (2002). An anti-rheumatic agent T-614 inhibits NF-kappaB activation in LPS- and TNF-alpha-stimulated THP-1 cells without interfering with IkappaBalpha degradation.. Inflamm Res.

[46] Akamatsu S, Takata R, Ashikawa K, Hosono N, Kamatani N (2010). A functional variant in NKX3.1 associated with prostate cancer susceptibility down-regulates NKX3.1 expression.. Hum Mol Genet.

[47] Johnson AD, Handsaker RE, Pulit SL, Nizzari MM, O'Donnell CJ (2008). SNAP: a web-based tool for identification and annotation of proxy SNPs using HapMap.. Bioinformatics.

