# Supplementary figures and images for: A Genome-Wide Association Study Identified AFF1 as a Susceptibility Locus for Systemic Lupus Eyrthematosus in Japanese
Source: PLoS Genet. 2012 Jan 26;8(1):e1002455. doi: 10.1371/journal.pgen.1002455 (PMC3266877; doi:10.1371/journal.pgen.1002455)

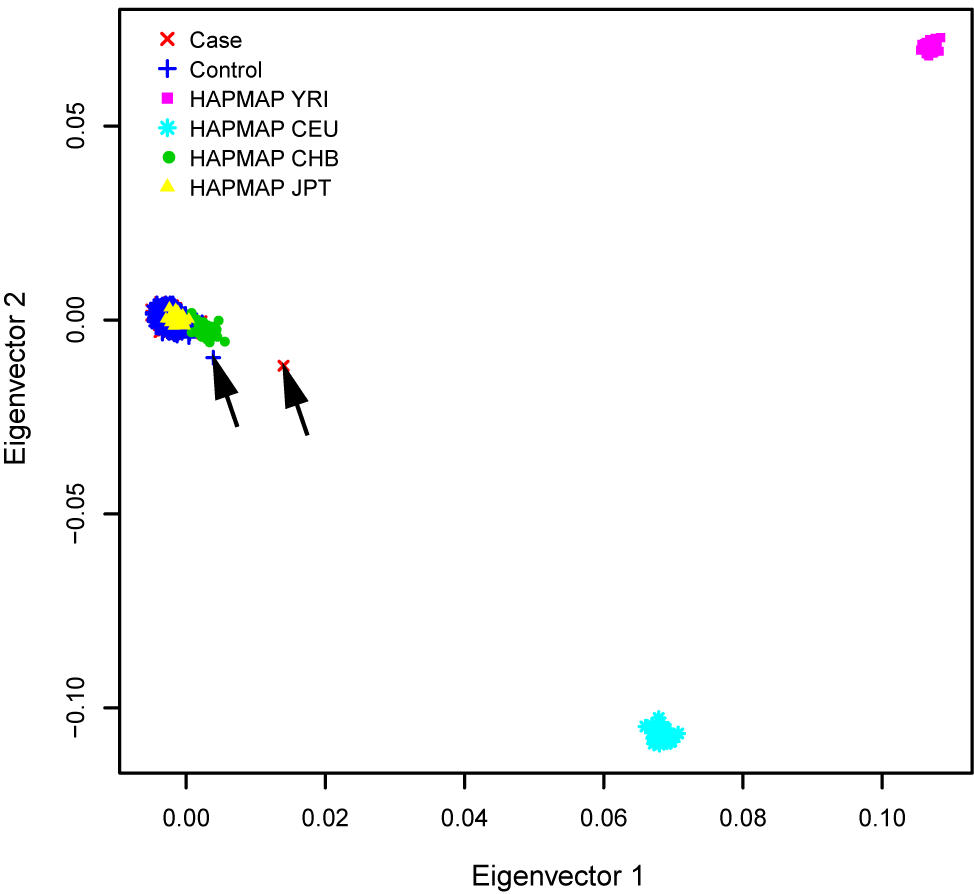

Supplement: Figure S1 — Principal component analysis (PCA) plot of the subjects. PCA plot of subjects enrolled in the GWAS for SLE. SLE cases and the controls enrolled in the GWAS are plotted based on eigenvectors 1 and 2 obtained from the PCA using EIGENSTRAT version 2.0 [42], along with European (CEU), African (YRI), Japanese (JPT), and Chinese (CHB) individuals obtained from the Phase II HapMap database (release 22) [29]. Subjects who were estimated to be outliers in terms of ancestry from East-Asian (JPT+CHB) clusters and excluded from the study are indicated by black arrows. (TIF) [file pgen.1002455.s001.tif]

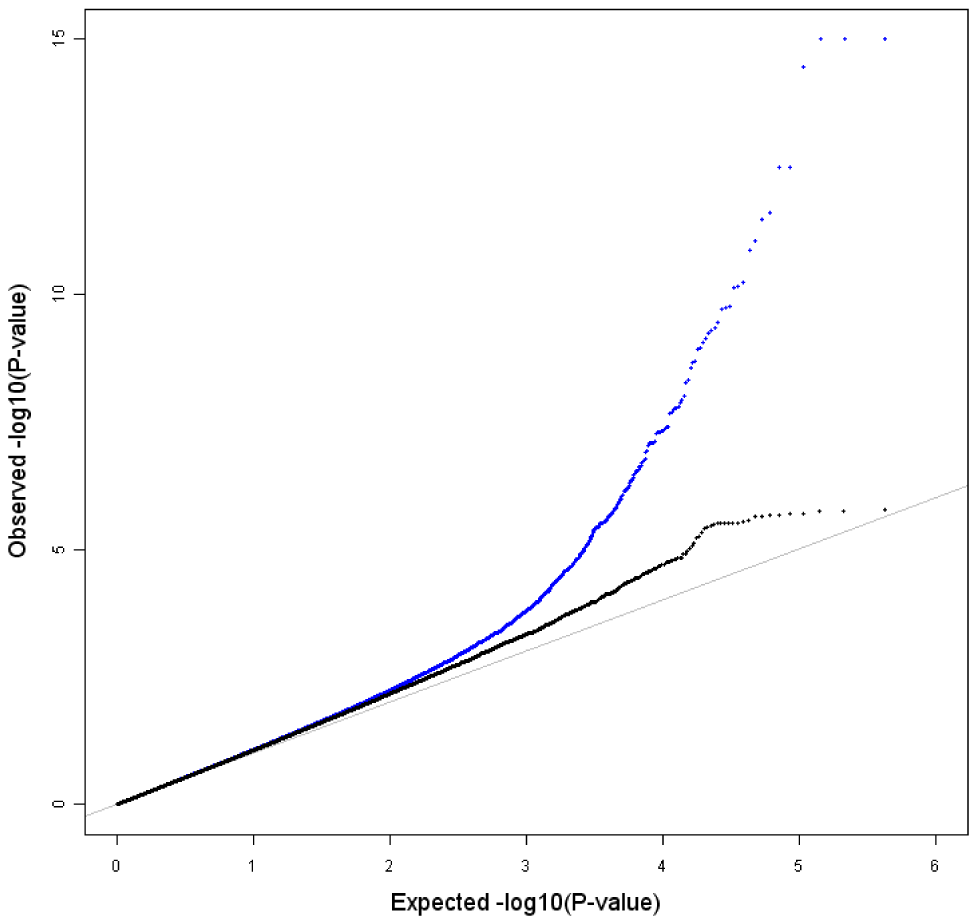

Supplement: Figure S2 — Quantile-Quantile plot (QQ-plot) of P-values in the GWAS for SLE. The horizontal axis indicates the expected −log10 (P-values). The vertical axis indicates the observed −log10 (P-values). The QQ-plot for the P-values of all SNPs that passed the quality control criteria is indicated in blue. The QQ-plot for the P-values after the removal of SNPs included in the previously reported SLE susceptibility loci is indicated in black. The gray line represents y = x. The SNPs for which the P-value was smaller than 1.0×10^−15^ are indicated at the upper limit of the plot. (TIF) [file pgen.1002455.s002.tif]
