# Supplementary material for: A Genome-Wide Association Study Identified AFF1 as a Susceptibility Locus for Systemic Lupus Eyrthematosus in Japanese
Source: PLoS Genet. 2012 Jan 26;8(1):e1002455. doi: 10.1371/journal.pgen.1002455 (PMC3266877; doi:10.1371/journal.pgen.1002455)
Supplement: Table S1 — Basal characteristics of cohorts. (DOC) [file pgen.1002455.s003.doc]

**Table S1. Basal characteristics of cohorts.**

| Disease | Study stage | Source | Genotyping platform | No. samples | Female | Age(mean ± sd) |
| --- | --- | --- | --- | --- | --- | --- |
| SLE cases^a^ | GWAS | ADSG | Illumina HumanHap610-Quad | 891 | 88.1% | 43.6 ± 13.9 |
|  | Replication study 1 | Kyushu University | Taqman genotyping system | 562 | 92.7% | NA |
|  | Replication study 2 | Kyoto University, Tokyo Women's Medical University | Taqman genotyping system | 825 | 92.3% | 40.3 ± 13.3 |
|  |  | University of Tokyo, ADSG |  |  |  |  |
|  |  |  |  |  |  |  |
|  | GWAS | BioBank Japan Project | Illumina HumanHap550v3 | 3,384 | 44.4% | 52.5±15.2 |
| Control subjects^a^ | Replication study 1 | Kyushu University | Taqman genotyping system | 653 | 81.2% | NA |
|  | Replication study 2 | BioBank Japan Project | Illumina HumanHap610-Quad | 27,911 | 43.6% | 63.1±12.1 |

^a^SLE cases enrolled in the GWAS (*n* = 891) or part of the 2nd replication study (*n* = 83) were collected from 12 medical institutes in Japan under the support of the Autoimmune Disease Study Group of Research in Intractable Diseases, Japanese Ministry of Health, Labor and Welfar. Japan (ADSG) : Hokkaido University Graduate School of Medicine, Tohoku University Graduate School of Medicine, the University of Tokyo, Keio University School of Medicine, Juntendo University School of Medicine, University of Occupational and Environmental Health, University of Tsukuba, Tokyo Medical and Dental University, National Center for Global Health and Medicine, Nagasaki University, Wakayama Medical University, and Jichi Medical University. SLE cases (*n* = 562) and controls (*n* = 653) enrolled in the 1st replication study were collected from Kyushu University. Some of the SLE cases (*n* = 742) and controls (*n* = 27,911) enrolled in the 2nd replication study were collected from Kyoto University, Tokyo Women's Medical University, the University of Tokyo, and the BioBank Japan Project.

SLE, systemic lupus erythematosus; GWAS, genome-wide association study; ADSG, Autoimmune Disease Study Group of Research in Intractable Diseases, Ministry of Health, Labor and Welfare, Japan; NA, not available.
