# Supplementary material for: A Genome-Wide Association Study Identified AFF1 as a Susceptibility Locus for Systemic Lupus Eyrthematosus in Japanese
Source: PLoS Genet. 2012 Jan 26;8(1):e1002455. doi: 10.1371/journal.pgen.1002455 (PMC3266877; doi:10.1371/journal.pgen.1002455)
Supplement: Table S2 — Frequency of clinical characteristics of SLE in this GWAS. (DOC) [file pgen.1002455.s004.doc]

**Table S2.** Frequency of clinical characteristics of SLE in this GWAS.

| Phenotypes | No. of positive / negative patients |
| --- | --- |
|  | (positive rate %) |
| Immunologic disorder | 737 / 38 (95.1) |
| Hematologic disorder | 708 / 48 (93.7) |
| Arthritis | 615 / 148 (80.6) |
| Malar rash | 520 / 239 (68.5) |
| Photosensitivity | 474 / 268 (63.9) |
| Renal disorder | 470 / 305 (60.6) |
| Oral ulcers | 288 / 442 (39.5) |
| Discoid rash | 201 / 535 (27.3) |
| Serositis | 197 / 545 (26.5) |
| Neurologic disorder | 152 / 592 (20.4) |
