# Supplementary material for: A Genome-Wide Association Study Identified AFF1 as a Susceptibility Locus for Systemic Lupus Eyrthematosus in Japanese
Source: PLoS Genet. 2012 Jan 26;8(1):e1002455. doi: 10.1371/journal.pgen.1002455 (PMC3266877; doi:10.1371/journal.pgen.1002455)
Supplement: Table S3 — Distributions of eQTL positivity rates of the SNPs. (DOC) [file pgen.1002455.s005.doc]

**Table S3.** Distributions of eQTL positivity rates of the SNPs.

| SNP definition | No. SNPs | No. eQTL positive SNPs^a^ | eQTL positivity rates (%)^a^ |
| --- | --- | --- | --- |
| SNPs in the SLE susceptibility loci | 26 | 8 | 30.8 |
| SNPs located ± 1 kbp of the probe | 5,921 | 902 | 15.2 |
| SNPs located ± 2.5 kbp of the probe | 13,811 | 2,010 | 14.6 |
| SNPs located ± 5 kbp of the probe | 25,603 | 3,556 | 13.9 |
| SNPs located ± 10 kbp of the probe | 46,008 | 6,048 | 13.1 |
| SNPs located ± 25 kbp of the probe | 95,216 | 11,035 | 11.6 |
| SNPs located ± 50 kbp of the probe | 153,031 | 15,493 | 10.1 |
| SNPs located ± 100 kbp of the probe | 222,876 | 19,209 | 8.6 |
| SNPs located ± 200 kbp of the probe | 288,483 | 21,321 | 7.4 |
| SNPs located ± 300 kbp of the probe | 317,554 | 21,786 | 6.9 |

^a^Estimated using gene expression data measured in lymphoblastoid B cell lines [28].

SLE, systemic lupus erythematosus; eQTL, expression quantitative trait locus.
