# Supplementary material for: A Genome-Wide Association Study Identified AFF1 as a Susceptibility Locus for Systemic Lupus Eyrthematosus in Japanese
Source: PLoS Genet. 2012 Jan 26;8(1):e1002455. doi: 10.1371/journal.pgen.1002455 (PMC3266877; doi:10.1371/journal.pgen.1002455)
Supplement: Table S4 — Results of replication study 1 for Japanese patients with SLE. (DOC) [file pgen.1002455.s006.doc]

**Table S4.** Results of replication study 1 for Japanese patients with SLE.

| rsID | Chr | Position (bp) | Cytoband | Gene | Allele^a^ | Stage | No. subjects | | Allele 1 freq. | | OR (95%CI) | *P* | eQTL^b^ |
| --- | --- | --- | --- | --- | --- | --- | --- | --- | --- | --- | --- | --- | --- |
|  |  |  |  |  | 1/2 |  | Case | Control | Case | Control |  |  |  |
|  |  |  |  |  |  | GWAS | 891 | 3,384 | 0.092 | 0.067 | 1.40 (1.16-1.69) | 3.5×10^-4^ |  |
| rs7591615 | 2 | 215,372,636 | 2q35 | *BARD1* | T/C | Replication study 1 | 550 | 637 | 0.062 | 0.068 | 0.90 (0.65-1.25) | 0.53 | + |
|  |  |  |  |  |  | Combined study | 1,441 | 4,021 | 0.080 | 0.068 | 1.25 (1.06-1.47) | 0.0068 |  |
|  |  |  |  |  |  | GWAS | 891 | 3,383 | 0.56 | 0.51 | 1.22 (1.10-1.36) | 1.5×10^-4^ |  |
| rs340630 | 4 | 88,177,419 | 4q21 | *AFF1* | A/G | Replication study 1 | 550 | 646 | 0.57 | 0.49 | 1.40 (1.19-1.64) | 4.6×10^-5^ | + |
|  |  |  |  |  |  | Combined study | 1,441 | 4,029 | 0.57 | 0.51 | 1.27 (1.17-1.39) | 6.9×10^-8^ |  |
|  |  |  |  |  |  | GWAS | 891 | 3,384 | 0.38 | 0.32 | 1.32 (1.18-1.47) | 9.4×10^-7^ |  |
| rs956237 | 4 | 109,266,409 | 4q25 | *LEF1* | A/G | Replication study 1 | 561 | 649 | 0.37 | 0.34 | 1.13 (0.95-1.33) | 0.16 |  |
|  |  |  |  |  |  | Combined study | 1,452 | 4,033 | 0.38 | 0.32 | 1.25 (1.15-1.37) | 9.4×10^-7^ |  |
|  |  |  |  |  |  | GWAS | 891 | 3,384 | 0.25 | 0.21 | 1.28 (1.13-1.44) | 1.0×10^-4^ |  |
| rs11132322 | 4 | 169,713,165 | 4q32 | *PALLD* | T/C | Replication study 1 | 560 | 652 | 0.20 | 0.24 | 0.82 (0.68-1.00) | 0.049 |  |
|  |  |  |  |  |  | Combined study | 1,451 | 4,036 | 0.23 | 0.21 | 1.12 (1.01-1.24) | 0.030 |  |
|  |  |  |  |  |  | GWAS | 891 | 3,384 | 0.74 | 0.79 | 0.77 (0.69-0.87) | 4.4×10^-5^ |  |
| rs7805536 | 7 | 32,540,095 | 7p14 | *KIAA0241* | T/C | Replication study 1 | 561 | 653 | 0.78 | 0.78 | 0.95 (0.78-1.15) | 0.58 |  |
|  |  |  |  |  |  | Combined study | 1,452 | 4,037 | 0.75 | 0.79 | 0.82 (0.74-0.91) | 1.3×10^-4^ |  |
|  |  |  |  |  |  | GWAS | 891 | 3,384 | 0.36 | 0.41 | 0.80 (0.72-0.89) | 6.7×10^-5^ |  |
| rs2979419 | 8 | 62,167,326 | 8q12 | *NPM1P6* | A/G | Replication study 1 | 556 | 648 | 0.40 | 0.39 | 1.02 (0.87-1.21) | 0.77 |  |
|  |  |  |  |  |  | Combined study | 1,447 | 4,032 | 0.38 | 0.41 | 0.86 (0.79-0.94) | 0.0013 |  |
|  |  |  |  |  |  | GWAS | 891 | 3,384 | 0.079 | 0.11 | 0.67 (0.55-0.80) | 1.7×10^-5^ |  |
| rs7302925 | 12 | 55,147,725 | 12q13 | *SPRYD4* | A/G | Replication study 1 | 558 | 649 | 0.10 | 0.11 | 0.87 (0.66-1.13) | 0.29 | + |
|  |  |  |  |  |  | Combined study | 1,449 | 4,033 | 0.086 | 0.11 | 0.72 (0.62-0.84) | 2.9×10^-5^ |  |
|  |  |  |  |  |  | GWAS | 891 | 3,373 | 0.66 | 0.71 | 0.82 (0.73-0.91) | 3.1×10^-4^ |  |
| rs220488 | 17 | 3,538,571 | 17p13 | *P2RX5* | A/G | Replication study 1 | 561 | 653 | 0.71 | 0.71 | 0.98 (0.82-1.17) | 0.80 | + |
|  |  |  |  |  |  | Combined study | 1,452 | 4,026 | 0.68 | 0.71 | 0.86 (0.78-0.95) | 0.0017 |  |

^a^Based on forward strand of NCBI Build 36.3.

^b^Defined using gene expression data measured in lymphoblastoid B cell lines [28].

SLE, systemic lupus erythematosus; OR, odds ratio; 95%CI, eQTL, expression quantitative trait locus; GWAS, genome-wide association study.
