# Supplementary material for: A Genome-Wide Association Study Identified AFF1 as a Susceptibility Locus for Systemic Lupus Eyrthematosus in Japanese
Source: PLoS Genet. 2012 Jan 26;8(1):e1002455. doi: 10.1371/journal.pgen.1002455 (PMC3266877; doi:10.1371/journal.pgen.1002455)
Supplement: Table S5 — Results of replication studies 1 and 2 for Japanese patients with SLE. (DOC) [file pgen.1002455.s007.doc]

**Table S5.** Results of replication studies 1 and 2 for Japanese patients with SLE.

| rsID | Chr | Position (bp) | Cytoband | Gene | Allele^a^ | Stage | No. subjects | | Allele 1 freq. | | OR (95%CI) | *P* | eQTL^b^ |
| --- | --- | --- | --- | --- | --- | --- | --- | --- | --- | --- | --- | --- | --- |
|  |  |  |  |  | 1/2 |  | Case | Control | Case | Control |  |  |  |
| rs340630 | 4 | 88,177,419 | 4q21 | *AFF1* | A/G | GWAS | 891 | 3,383 | 0.56 | 0.51 | 1.22 (1.10-1.36) | 1.5×10^-4^ | + |
|  |  |  |  |  |  | Replication study 1 | 550 | 646 | 0.57 | 0.49 | 1.40 (1.19-1.64) | 4.6×10^-5^ |  |
|  |  |  |  |  |  | Replication study 2 | 820 | 27,911 | 0.56 | 0.53 | 1.14 (1.03-1.26) | 0.0094 |  |
|  |  |  |  |  |  | Combined study | 2,261 | 31,940 | 0.56 | 0.52 | 1.21 (1.14-1.30) | 8.3×10^-9^ |  |
| rs956237 | 4 | 109,266,409 | 4q25 | *LEF1* | A/G | GWAS | 891 | 3,384 | 0.38 | 0.32 | 1.32 (1.18-1.47) | 9.4×10^-7^ |  |
|  |  |  |  |  |  | Replication study 1 | 561 | 649 | 0.37 | 0.34 | 1.13 (0.95-1.33) | 0.16 |  |
|  |  |  |  |  |  | Replication study 2 | 742 | 27,907 | 0.35 | 0.33 | 1.09 (0.99-1.21) | 0.085 |  |
|  |  |  |  |  |  | Combined study | 2,194 | 31,940 | 0.37 | 0.33 | 1.18 (1.10-1.26) | 1.5×10^-6^ |  |

^a^Based on forward strand of NCBI Build 36.3.

^b^Defined using gene expression data measured in lymphoblastoid B cell lines [28].

SLE, systemic lupus erythematosus; OR, odds ratio; 95%CI, eQTL, expression quantitative trait locus; GWAS, genome-wide association study.
